# Complementary and Alternative Medicine for the Treatment of Fibromyalgia

**DOI:** 10.1155/2014/408436

**Published:** 2014-01-09

**Authors:** Jost Langhorst, Winfried Häuser, Romy Lauche, Serge Perrot, Cayetano Alegre, Piercarlo C. Sarzi Puttini

**Affiliations:** ^1^Department of Internal and Integrative Medicine, Kliniken Essen-Mitte, Faculty of Medicine, University of Duisburg-Essen, 45276 Essen, Germany; ^2^Department of Internal Medicine 1, Klinikum Saarbrücken, 66119 Saarbrücken, Germany; ^3^Department of Psychosomatic Medicine and Psychotherapy, Technische Universität München, 81865 München, Germany; ^4^Service de Médecine Interne et Thérapeutique, Centre de la Douleur, Hôtel Dieu, Université Paris Descartes, INSERM U 987, Paris, France; ^5^Department of Rheumatology, Hospital Universitari Vall d'Hebron, Barcelona, Spain; ^6^Rheumatology Unit, L Sacco University Hospital, Milan, Italy

The fibromyalgia syndrome (FMS) is a chronic condition characterized by chronic widespread pain, fatigue, cognitive disturbances, sleep disorders, and somatic and psychological distress [1, 2]. Between 2.9 and 3.8% of the general population in Europe and the US are affected [3–5], with 9 times as many women as men in clinical settings [2].

Many patients with fibromyalgia use complementary and alternative therapies to cope with their disease. A recent consumers report indicated that 67.0% of German FMS patients used heat application or thermal baths, 35.2% different CAM medications such as homeopathy, dietary supplements, and vitamins, 34.6% some kind of diet, 28.5% tool-based physical therapies such as acupuncture, and 18.4% meditative exercises such as yoga or tai chi [6]. In summary, almost every FMS patient had used at least one CAM therapy for the management of FMS. In an Internet sample of US American FMS patients, the frequencies were slightly higher [7]. CAM use in FMS patients is associated with younger age, female gender, and higher overall disease burden [8, 9].

Contrary to the frequent use, only limited research has been conducted on complementary and alternative therapies so far. Data on efficacy and safety are however necessary to judge their value within the treatment regimen. Without reliable information, such therapies that might benefit FMS patients will also not be included in standard care; therapies with a negative benefit-risk ratio on the other hand might be applied despite doubts about their effects.

This special issue aimed to facilitate publication of research of complementary and alternative therapies for the fibromyalgia syndrome. The papers published in this special issue were carefully selected to display a great variety of different research topics. In the following, a short overview of included papers will be provided.

M.-A. Fitzcharles et al. took a closer look at classification and clinical diagnosis of the fibromyalgia syndrome. Three evidence-based interdisciplinary guidelines from Canada, Germany, and Israel were compared in terms of definition and diagnostic procedures, and consistent results have been found for all three guidelines. The results of this review further implied the importance of evidence-based guidelines for health care providers. J. N. Ablin et al. summarized the recommendations of those guidelines with a special emphasis on CAM therapies. While all guidelines supported patient-tailored approaches, recommendations differed, especially regarding CAM. Discrepancies were also present regarding the levels of evidence and the strengths of recommendation. Contrary to Germany and Israel, where some CAM therapies were recommended, no CAM treatment was recommended according to the Canadian guidelines. This supports the need for high-quality research.

Three papers reported on clinical trials. In the so-called KAFA-trial, C. S. Kessler et al. investigated the feasibility and efficacy of an additive complex Ayurvedic treatment in 32 FMS patients, using a nonrandomized controlled study design. All patients received conventional care during an inpatient hospital admission with elements of physiotherapy, hydrotherapy, exercise, and cognitive behavioral and occupational therapies. The Ayurvedic group also received individual treatment regimen including massages, diets, yoga, and self-help strategies. At the end of two weeks, different parameters including quality of life, pain, and sleep quality were assessed. A prospective observational trial by K. Kraft et al. with 70 patients tested the effects of vibration massage using a deep oscillation device. Patients were treated twice weekly for five weeks and followed up for two months. Outcomes included safety and tolerability, symptom severity, and quality of life. Finally, J. Sawynok et al. reported the results of an extension trial that investigated 20 FMS patients who had been included in a trial on the effects of qigong for six months. Outcomes included quantitative measures such as pain intensity, disability and quality of life, and qualitative comments of participants.

Another four manuscripts reported the results of reviews; one of them was a narrative review and three were systematic reviews, partially with meta-analyses. J. N. Ablin et al. provided a qualitative-narrative review and a historical perspective on the use of spa treatment for fibromyalgia. The authors not only included clinical trials, but also publications regarding the history, the implementation of spa therapy, and related practices in different cultures and provided the readers with a comprehensive overview. Another review by S. S. Nascimento et al. systematically reviewed the efficacy and safety of medicinal plants or related natural products for fibromyalgia. Eight randomized controlled trials were included in this analysis; possible benefits and harms were investigated. R. Lauche et al. aimed to summarize the efficacy and safety of qigong trials. Seven randomized controlled trials with 395 were included and the authors analyzed whether quality of life and other key symptoms were altered by qigong interventions. An established tool to formulate recommendation for clinical practice was also applied. And J. A. Glombiewski et al. conducted a systematic review on EMG and EEG-Biofeedback for FMS. Seven randomized controlled trials were included based on 321 patients. Together these reviews provide up to date information about the state of scientific evidence for the respective topics.

The authors are confident that this special issue will provide readers with an insightful cross-section of current FMS research topics and facilitate further high-quality research in the CAM field for the sake of FMS patients.

*Jost Langhorst* *Jost Langhorst* *Winfried Häuser* *Winfried Häuser* *Romy Lauche* *Romy Lauche* *Serge Perrot* *Serge Perrot* *Cayetano Alegre* *Cayetano Alegre* *Piercarlo C. Sarzi Puttini* *Piercarlo C. Sarzi Puttini*
